# NKLP27: A Teleost NK-Lysin Peptide that Modulates Immune Response, Induces Degradation of Bacterial DNA, and Inhibits Bacterial and Viral Infection

**DOI:** 10.1371/journal.pone.0106543

**Published:** 2014-09-02

**Authors:** Min Zhang, Mo-fei Li, Li Sun

**Affiliations:** 1 Key Laboratory of Experimental Marine Biology, Institute of Oceanology, Chinese Academy of Sciences, Qingdao, China; 2 College of Marine Science and Engineering, Qingdao Agricultural University, Qingdao, China; 3 Collaborative Innovation Center of Deep Sea Biology, Zhejiang University, Hangzhou, China; 4 Graduate University of the Chinese Academy of Sciences, Beijing, China; Friedrich-Loeffler Institut, Germany

## Abstract

NK-lysin is an antimicrobial protein produced by cytotoxic T lymphocytes and natural killer cells. In this study, we examined the biological property of a peptide, NKLP27, derived from tongue sole (*Cynoglossus semilaevis*) NK-lysin. NKLP27 is composed of 27 amino acids and shares little sequence identity with known NK-lysin peptides. NKLP27 possesses bactericidal activity against both Gram-negative and Gram-positive bacteria including common aquaculture pathogens. The bactericidal activity of NKLP27 was dependent on the C-terminal five residues, deletion of which dramatically reduced the activity of NKLP27. During its interaction with the target bacterial cells, NKLP27 destroyed cell membrane integrity, penetrated into the cytoplasm, and induced degradation of genomic DNA. In vivo study showed that administration of tongue sole with NKLP27 before bacterial and viral infection significantly reduced pathogen dissemination and replication in tissues. Further study revealed that fish administered with NKLP27 exhibited significantly upregulated expression of the immune genes including those that are known to be involved in antibacterial and antiviral defense. These results indicate that NKLP27 is a novel antimicrobial against bacterial and viral pathogens, and that the observed effect of NKLP27 on bacterial DNA and host gene expression adds new insights to the action mechanism of fish antimicrobial peptides.

## Introduction

In the past years, wide spread use of traditional antibiotics in aquaculture has led to the emergence of diverse multidrug-resistant bacterial strains that pose serious threats to public health [1]–[3]. As such, it is imperative to find new antimicrobials for the control of aquaculture diseases. At present, antimicrobial peptides (AMPs) have attracted increasing interest for their broad-spectrum antimicrobial activities and, most importantly, their unique mode of action that prevents the development of resistant variants [4]–[8]. AMPs are short, cationic peptides with an amphipathic secondary structure, by which AMPs destruct target cell membrane rapidly without the need of interaction with specific receptors [4], [8]. AMPs are widespread in nature, and so far more than 1,800 AMPs have been discovered [9], [10]. In fish, a large number of AMPs have been identified in recent years, including hepcidin, defensins, pleurocidin, piscidin, moronecidin, misgurain, parasin, LEAP-2, and NK-lysin [11]–[20].

NK-lysin is a type of AMP that is produced by cytotoxic T lymphocytes and nature killer cells [21]. NK-lysin homologues have been identified in a variety of organisms, some of which possess antibacterial activity against Gram-negative and/or Gram-positive bacteria [22]–[27]. However, the length of the NK-lysin protein (78 residues) makes it difficult for chemical synthesis or biosynthesis. Therefore, short peptides derived from NK-lysin with antimicrobial effect have been studied. For example, NK-2, a derivative of porcine NK-lysin, is a 27-residue AMP against *Candida albicans*, bacteria, and *Trypanosoma cruzi* [28], [29]; NK-18, a shortened analogue of NK-2, shows potent antibacterial and antitumor activity against bladder and prostate cancer cells [8], [30]. However, in fish, similar studies on NK-lysin peptides are scarce, and to date the only documented report is on the function of a synthesized peptide derived from flounder NK-lysin, which displays antimicrobial activity against several Gram-negative bacteria, but the action mechanism of the peptide is unknown [20].

In a previous study, we identified a NK-lysin gene (named *CsNKL1*) from half-smooth tongue sole (*Cynoglossus semilaevis*) and found that *in vivo* overexpression of CsNKL1 inhibited bacterial and viral infection [31]. In the present study, we synthesized a peptide, CsNKLP27, based on the sequence of CsNKL1 and investigated its antimicrobial potential and action mode. We found that NKLP27 is a novel AMP that induced degradation of bacterial DNA and blocked the invasion of not only pathogenic bacteria but also virus. In addition, we also observed an immunoregulatory effect of NKLP27.

## Materials and Methods

### Ethics statement

Experiments involving live animals were conducted in accordance with the “Regulations for the Administration of Affairs Concerning Experimental Animals” promulgated by the State Science and Technology Commission of Shandong Province. The study was approved by the ethics committee of Institute of Oceanology, Chinese Academy of Sciences.

### Fish

Clinically healthy half-smooth tongue sole (*Cynoglossus semilaevis*) (average 11.2±0.1 g) were purchased from a commercial fish farm in Shandong Province, China and maintained at 20°C in aerated seawater. Before experimental manipulation, fish were randomly sampled for the examination of the presence of bacteria and megalocytivirus in blood, liver, kidney, and spleen as reported previously [32], [33], and no pathogens were detected in these tissues. Fish were euthanized with an overdose of tricaine methanesulfonate (Sigma, St. Louis, MO, USA) prior to experiments involving tissue collection [34].

### Bacterial strains and culture conditions

The fish pathogens *Pseudomonas fluorescens* TSS, *Vibrio/Listonella anguillarum* C312, *Vibrio harveyi* T4, and *Streptococcus iniae* SF1 have been reported previously [34]–[36]. *Escherichia coli* DH5α was purchased from Tiangen (Beijing, China); *Micrococcus luteus* and *Staphylococcus aureus* were purchased from China General Microbiological Culture Collection Center (Beijing, China). *S. iniae* SF1 was cultured in TSAYE medium at 28°C, all other strains were cultured in Luria-Bertani broth (LB) medium either at 37°C (for *E. coli*, *M. luteus* and *S. aureus*) or at 28°C (for *P. fluorescens*, *S. iniae*, *V. anguillarum*, and *V. harveyi*).

### Peptides

Unlabeled and 5′-FAM labeled NKLP27 (KVKARLIKICNKIGFLKSRCHKFVITH) and NKLP22 (KVKARLIKICNKIGFLKSRCHK) were chemically synthesized with an amidated C-terminus by Sangon (Shanghai, China). The control peptide P86P15 [37] was synthesized similarly. The peptides were purified by high-performance liquid chromatography to >90% of purity. Lyophilized peptides were stored at −20°C and dissolved in PBS (pH 6.5) before use.

### Minimum inhibitory concentration (MIC) and minimal bactericidal concentration (MBC) assays

*E. coli*, *V. anguillarum*, *V. harveyi, P. fluorescence, M. luteus, S. iniae*, and *S. aureus* were cultured as above to mid-logarithmic phase. The cells were centrifuged, washed, and resuspended in PBS to 2×10^5^ CFU/ml. NKLP27, NKLP22, and P86P15 were dissolved in PBS and diluted serially in two-fold. The dilution (1 µl each) was mixed with 100 µl of bacterial culture. For MIC assay, the mixture was incubated at 28°C or 37°C (as indicated above for different bacteria) for 24 h and then inspected for growth. MIC was defined as the lowest peptide concentration that prevented visible growth. For MBC assay, the mixture was incubated at 28°C or 37°C for 24 h; after incubation, the mixture was diluted and plated in triplicate on TSAYE (for *S. iniae*) or LB (for all other bacteria) agar plates. The plates were incubated at 28°C or 37°C (as indicated above for different bacteria) for 48 h, and the colonies that appeared on the plates were counted. MBC was defined as the lowest peptide concentration that resulted in no colony emergence on the plates. The assays were performed three times.

### Propidium iodide (PI) uptake assay

*V. anguillarum* was cultured as above and resuspended in PBS to 5×10^8^ CFU/ml. One hundred microliters of bacterial cells were incubated with NKLP27 or P86P15 (3 µM, 6 µM, 12 µM, and 24 µM) at 28°C for 1 h, and the cells were then stained with PI solution (20 µg/ml) for 30 min at room temperature in the dark. After the reaction, the cells were centrifuged and washed with PBS. The ratio of PI-stained cells was determined with a flow cytometer (Beckman Coulter, USA).

### Electron microscopy

*V. anguillarum* was cultured in LB medium to mid-logarithmic phase and resuspended in PBS to 5×10^8^ CFU/ml. One hundred microliters of bacterial cells were mixed with 6 µM NKLP27, and the mixture was incubated at 28°C for 2 h or 4 h. After incubation, the cells were fixed with glutaraldehyde and deposited on carbon-coated copper grids. The grids were dried naturally and negative stained with phosphotungstic acid. The grids were then observed with a transmission electron microscope (GEM-1200, GEOL, Japan).

### Fluorescence microscopy

*V. anguillarum* was cultured in LB medium to mid-logarithmic phase and resuspended in PBS to 5×10^8^ CFU/ml. Ten microliters of bacterial cells were incubated with 6 µM FAM-labeled NKLP27 or P86P15 for 0.5 h. After incubation, the cells were washed with PBS to remove unbound peptide. Extracellular fluorescence was quenched by adding 0.4% trypan blue into the cells, followed by incubation of the cells at room temperature for 1 h. After incubation, the cells were centrifuged and washed with PBS. The cells were immobilized on a glass slide and observed with a fluorescence microscope (Nikon E800, Japan).

### Effect of NKLP27 on DNA

For *in vitro* assay, genomic DNA (100 ng) of *V. anguillarum* was mixed with different concentrations of NKLP27 or P86P15 in a total volume of 20 µl PBS. The mixture was incubated at room temperature for 30 min and subjected to agarose gel electrophoresis. For *in vivo* assay, *V. anguillarum* was cultured and resuspended in PBS to 5×10^8^ CFU/ml as described above. One hundred microliters of the bacterial suspension was mixed with 6 µM NKLP27 or P86P15. The mixture was incubated at 28°C for 2 h, 4 h, or 6 h. After incubation, genomic DNA was extracted with TIANamp Bacteria DNA Kit (Tiangen, Beijing, China) and subjected to agarose gel electrophoresis.

### Quantitative real time reverse transcriptase-PCR (qRT-PCR) analysis of gene expression

Tongue sole as described above were randomly divided into three groups and administered via intraperitoneal injection with 50 µl PBS containing 6.25 µM NKLP27 or P86P15. The control group was injected with 50 µl PBS. At 1 h, 12 h, and 24 h post-injection, tissues were collected and used for total RNA extraction with the RNAprep Tissue Kit (Tiangen, Beijing, China). One microgram of RNA was used for cDNA synthesis with the Superscript II reverse transcriptase (Invitrogen, Carlsbad, USA). The expression levels of interleukin (IL)-1β, IL-8, CsCCK1, CsCXCe1, toll-like receptor 9 (TLR9), myeloid differentiation factor 88 (Myd88), CsISG15, and CsG3BP were determined by qRT-PCR with primers reported previously [9], [38]–[40]. qRT-PCR was carried out in an Eppendorf Mastercycler (Eppendorf, Hamburg, Germany) using the SYBR ExScript qRT-PCR Kit (Takara, Dalian, China) as described previously [41]. Melting curve analysis of amplification products was performed at the end of each PCR to confirm that only one PCR product was amplified and detected. The expression levels of the immune genes were analyzed using comparative threshold cycle method with beta-actin as the control.

### Effect of NKLP27 on pathogen infection

Tongue sole were administered with or without NKLP27 or P86P15 as described above. At 1 h post-peptide administration, the fish were inoculated via intraperitoneal injection with *V. anguillarum* (10^5^ CFU/fish) or megalocytivirus RBIV-C1 [33] (10^4^ copies/fish). Tissues were taken under aseptic conditions at 12 h and 24 h after bacterial infection or at 3 d and 5 d after viral infection. To examine bacterial loads in the tissues, the tissues were homogenized in PBS, and the homogenates were diluted in PBS and plated in triplicate on LB agar plates. The plates were incubated at 28°C for 48 h, and the colonies that emerged on the plates were counted. The genetic nature of the colonies was verified by PCR and sequence analysis of the PCR products. Viral copy number in the tissues was determined by absolute quantitative real time PCR as reported previously [33].

### Statistical analysis

All statistical analyses were performed with SPSS 17.0 software (SPSS Inc., Chicago, IL, USA). Data were analyzed with one-way analysis of variance (ANOVA), followed by Dunnett’s test. Statistical significance was defined as *P*<0.05.

## Results

### Bactericidal activity of NKLP27

Two small peptides, NKLP27 and NKLP22, were synthesized based on the sequence of CsNKL1. NKLP27 is composed of 27 residues that form the H2 and H3 α-helices of the SapB domain of CsNKL1 (positions 71–133 in CsNKL1), while NKLP22 is a truncated version of NKLP27 and lacks the last five residues of NKLP27. NKLP27 and NKLP22 have different molecular masses (3.2 kDa and 2.6 kDa respectively) but identical pI (10.7). Sequence alignment showed that NKLP27 shares 10.7% to 32.1% overall identities with synthetic peptides derived from fish, chicken, bovine, and porcine NK-lysin (Figure S1). To examine its potential antimicrobial effect, NKLP27 at different concentrations was incubated with the Gram-negative bacteria *E. coli*, *V. anguillarum*, *V. harveyi*, and *P. fluorescence,* and the Gram-positive bacteria *S. iniae*, *S. aureus*, and *M. luteus*. Of these bacteria, *V. anguillarum*, *V. harveyi*, *P. fluorescence,* and *S. iniae* are common fish pathogens. The results showed that for the examined bacterial species, the MIC values of NKLP27 ranged between 1 µM to 8 µM, with the lowest MIC value being that against *M. luteus*, and the highest MIC value being against *P. fluorescence* (Table 1). Similarly, the lowest and highest MBC values (4 µM and 32 µM respectively) were those against *M. luteus* and *P. fluorescence* respectively (Table 1). In contrast, treatment with P86P15, a peptide unrelated to CsNKL1, had no effect on the viability of the bacterial cells. Compared to NKLP27, NKLP22 exhibited a much weaker bactericidal effect. When *V. anguillarum* was used as a substrate strain, the MIC and MBC of NKLP22 were 32 µM and 128 µM respectively, which were much higher than those of NKLP27 (2 µM and 8 µM).

**Table 1:** Minimum inhibitory concentration (MIC) and minimum bactericidal concentration (MBC) of NKLP27 against Gram-negative and Gram-positive bacteria.

Strain	MIC (µM)	MBC (µM)
Gram-negative		
*Escherichia coli*	4	16
*Pseudomonas fluorescence*	8	32
*Vibrio anguillarum*	2	8
*Vibrio harveyi*	2	16
Gram-positive		
*Micrococcus luteus*	1	4
*Staphylococcus aureus*	4	16
*Streptococcus iniae*	4	16

### Effect of NKLP27 on the membrane integrity of the target cells

**Figure 1:**
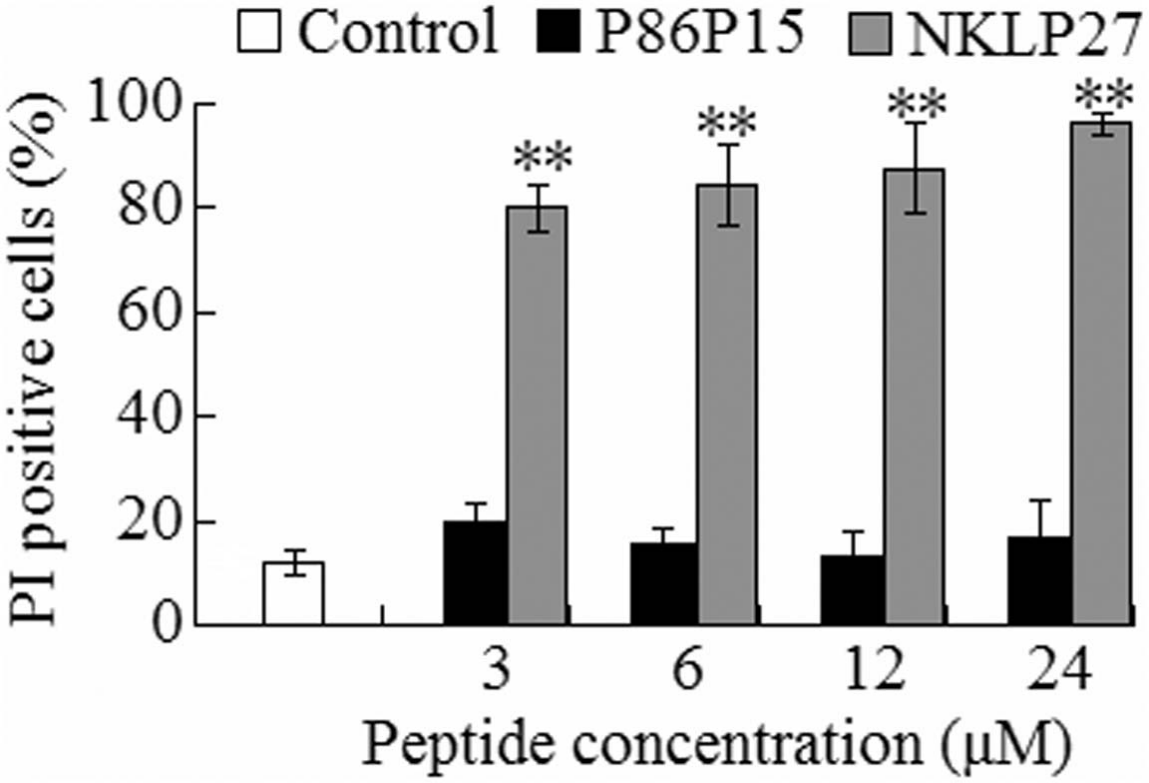
Effect of NKLP27 on the integrity of the target cell membrane. *Vibrio anguillarum* was incubated with different concentrations of NKLP27, P86P15, or PBS (control) for 1 h. The cells were stained with PI solution, and the percentage of PI-positive e cells was determined. The results are the means of three independent experiments and shown as means ± SE.

To examine whether NKLP27 affected the membrane integrity of the target cells, *V. anguillarum* was treated with NKLP27 at the doses of 3 µM, 6 µM, 12 µM, and 24 µM, and the cells were then subjected to PI staining assay. The results showed that the numbers of PI-positive cells increased with the concentration of NKLP27 and ranged between 80.1% to 96.3%, which were significantly higher than that of the control cells (Fig. 1). In contrast, the amounts of PI-positive cells in P86P15-treated *V. anguillarum* were comparable to that of the control.

### NKLP27-induced morphological change in the target cells

**Figure 2:**
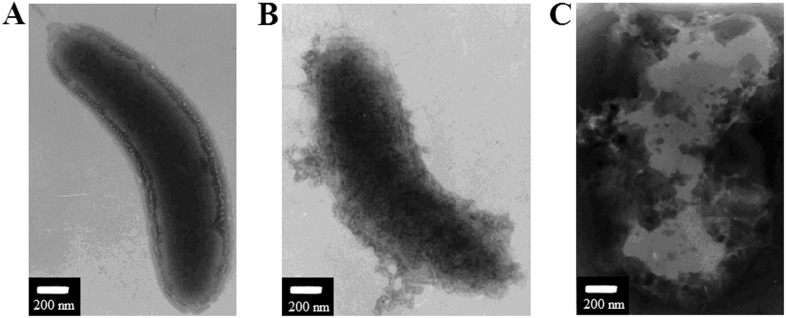
Effect of NKLP27 on the cell structure of *Vibrio anguillarum*. *V. anguillarum* was treated with NKLP27 for 2 h (B) or 4 h (C) and then subjected to electron microscopic examination. A, untreated control cell.

With above observation, we further examined whether NKLP27 had any effect on the general structure of the target bacterial cells. For this purpose, *V. anguillarum* treated with or without NKLP27 for different hours were subjected to electron microscopic examination. The results showed that the smooth layer of surface structure, which was displayed clearly by untreated control cells, was lost in cells treated with NKLP27 for 2 h (Fig. 2). At 4 h post-NKLP27 treatment, the cells displayed a completely damaged morphology, with leakage of cellular contents and breaking up of the basic cellular structure.

### Penetration of NKLP27 into the target cells

**Figure 3:**
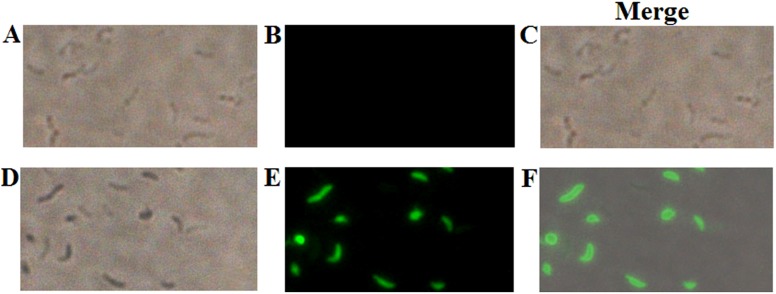
Penetration of NKLP27 into the target bacterial cells. *Vibrio anguillarum* was incubated with FAM-labeled NKLP27 (D and E) or the control peptide P86P15 (A and B) for 0.5 h. After quenching extracellular fluorescence, the cells were observed under a microscope with (B and E) or without (A and D) fluorescence. C, merged image of A and B; F, merged image of D and E. Magnification, 400×.

Since NKLP27 could disrupt the membrane and alter the structure of the target cells, it was possible that the peptide may enter into the cytoplasm. To examine this possibility, *V. anguillarum* was incubated with FAM-labeled NKLP27 or P86P15, and intracellular accumulation of the peptide was visualized by fluorescence microscopy. The results showed that NKLP27 was observed inside almost all cells, whereas no apparent intracellular P86P15 was observed (Fig. 3).

### Effect of NKLP27 on bacterial DNA

**Figure 4:**
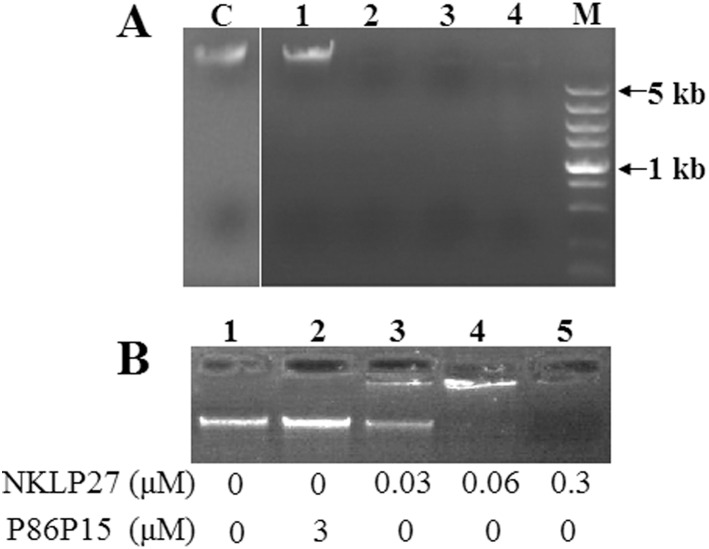
*In vivo* (A) and *in vitro* (B) effect of NKLP27 on bacterial DNA. (A) *Vibrio anguillarum* was incubated with NKLP27 for 0 h, 2 h, 4 h, and 6 h (lanes 1 to 4), or with P86P15 for 6 h (lane C), and genomic DNA was extracted and subjected to electrophoresis in an agarose gel. (B) The genomic DNA of *Vibrio anguillarum* was incubated with or without different concentrations of NKLP27 or P86P15. After incubation, the DNA was subjected to electrophoresis as above.

Given the ability of NKLP27 to enter into the target cells as observed above, which should enable direct contact of the peptide with the acidic genomic DNA of the target cells, we wondered whether NKLP27 had any effect on the DNA. To investigate this question, NKLP27 was incubated with *V. anguillarum* for various hours, and genomic DNA was extracted and subjected to electrophoresis. The results showed that incubation with NKLP27 for 2 h or longer time caused almost complete degradation of the DNA, whereas incubation with P86P15 had no apparent effect on the DNA (Fig. 4A). To examine the effect of NKLP27 *in vitro*, the genomic DNA of *V. anguillarum* was incubated with different concentrations of NKLP27 or P86P15, and the DNA was then subjected to electrophoresis. The results showed that incubation with 0.03 µM and 0.06 µM NKLP27 caused partial and complete blockage of DNA migration respectively, while incubation with 0.3 µM NKLP27 caused almost complete degradation of the DNA (Fig. 4B).

### Potential toxicity of NKLP27 to fish

Since, as shown in above results, NKLP27 possesses apparent antibacterial property, we wanted to investigate the potential of this peptide to be used *in vivo* in fish. For this purpose, we first examined whether NKLP27 had any toxic effect on fish. This was performed by administering into tongue sole 6.25 µM NKLP27 and monitoring the fish for growth and any pathological signs. The results showed that compared to untreated control fish, NKLP27-administered fish exhibited no detectable alterations in behavior, growth rate, weight, red blood cell count, or histological section within the one-month monitoring period. These results suggest that NKLP27 is probably nontoxic to fish.

### Effect of NKLP27 on bacterial and viral infection

**Figure 5:**
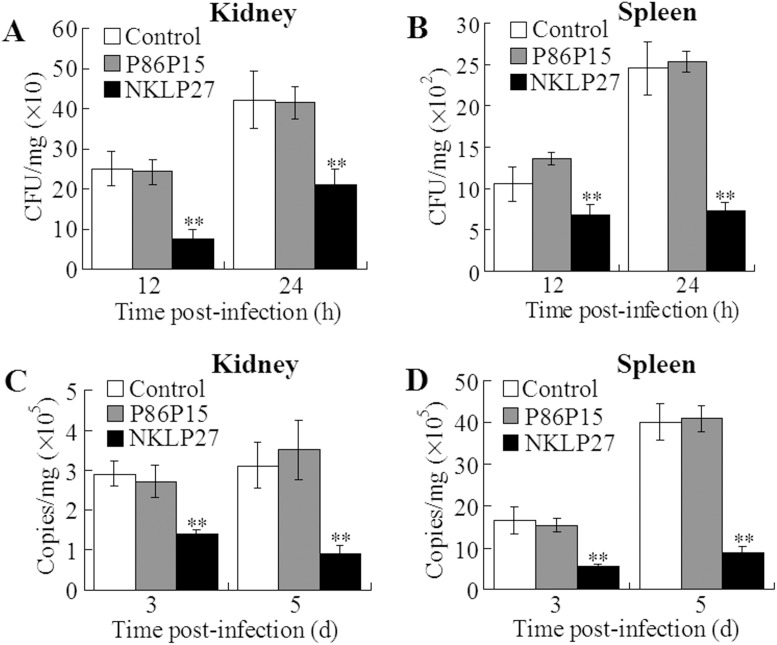
Effect of NKLP27 on bacterial and viral infection. Tongue sole were administered with or without (control) NKLP27 or P86P15 before being infected with *Vibrio anguillarum* (A and B) or megalocytivirus (C and D). The amounts of pathogens in kidney and spleen were determined at different time points post-infection. The results are the means of three independent experiments and shown as means ± SE. ^**^ *P*<0.01.

To examine the *in vivo* antimicrobial potential of NKLP27, tongue sole were administered with NKLP27 or P86P15 before being inoculated with *V. anguillarum* or the viral pathogen megalocytivirus. Pathogen burdens in the kidney and spleen of the infected fish were subsequently determined at different time points of infection. The results showed that at 12 h and 24 h post-infection, the numbers of *V. anguillarum* recovered from the kidney and spleen of NKLP27-administered fish were significantly lower than those from the control fish (Fig. 5A and B). In contrast, *V. anguillarum* recoveries from P86P15-administered fish were comparable to those from the control fish. Similarly, for fish infected with megalocytivirus, the viral loads in the kidney and spleen of NKLP27-administered fish were significantly lower than those in the control fish or in the fish administered with P86P15 (Fig. 5C and D).

### Effect of NKLP27 on immune gene expression

**Figure 6:**
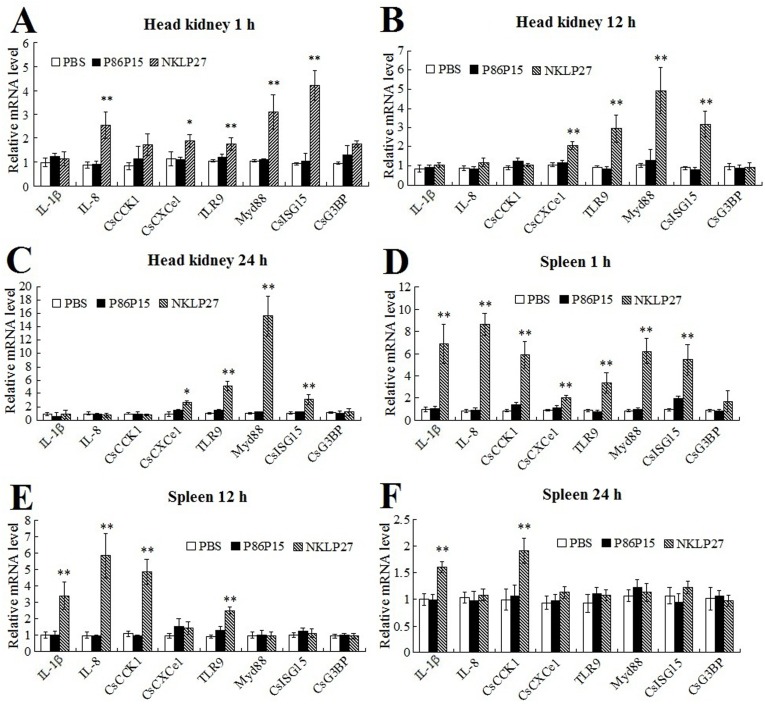
Effect of NKLP27 on immune gene expression. Tongue sole were administered with NKLP27, P86P15, or PBS (control). At 1 h, 12 h, and 24 h post-peptide administration, expression of the immune genes in head kidney and spleen was determined by quantitative real time RT-PCR. In each case, the expression level of the control fish was set as 1. The results are the means of three independent experiments and shown as means ± SE. ^**^ *P*<0.01.

With the above results, which indicated that NKLP27 possessed apparent antibacterial and antiviral properties, we wondered whether NKLP27 could affect the expression of the immune genes associated with antibacterial and antiviral defense. To investigate this question, we searched the available gene database of tongue sole and selected 8 genes, i.e., IL-1β, IL-8, CsCCK1 (a CC chemokine), CsCXCe1 (a CXC chemokine), TLR9, Myd88, CsISG15 (an ISG15 homologue), and CsG3BP, which are known to be involved in innate immune defense against bacterial and viral infection. The potential effect of NKLP27 on the expression of these genes in head kidney and spleen of tongue sole was examined by qRT-PCR at different time points after NKLP27 administration. The results showed that following administration of NKLP27, four genes, i.e., CsCXCe1, TLR9, Myd88, and CsISG15, were significantly upregulated in head kidney at all examined time points, one gene, i.e., IL-8, was significantly upregulated at 1 h post-peptide administration, whereas three genes, i.e., IL-1β, CsCCK1, and CsG3BP, exhibited no expressional change at all examined time points (Fig. 6A to C). In spleen, at 1 h post-NKLP27 administration, all genes except for CsG3BP were significantly upregulated; at 12 h post-NKLP27 administration, IL-1β, IL-8, CsCCK1, and TLR9 were significantly upregulated; at 24 h post-NKLP27 administration, only IL-1β and CsCCK1 were significantly upregulated (Fig. 6D to F).

## Discussion

In this study, we investigated the antimicrobial potential of a tongue sole NK-lysin peptide. Reports have shown that at least 12 synthetic peptides derived from the NK-lysin sequences of chicken and mammals possess antimicrobial activity against bacteria or parasites [20], [22], [24], [27]–[30], [42]–[44]. In fish, a synthesized peptide based on the sequence of flounder NK-lysin exerts killing effect on Gram-negative bacteria [20]. Similar to these observations, in our study, we found that NKLP27 exhibited bactericidal activity against both Gram-negative and Gram-positive bacteria. Compared to NKLP27, the C-terminally truncated NKLP22 was dramatically reduced in activity. Previous studies showed that NK-lysin peptides with AMP properties all locate in the SapB domain and incorporate several α-helices, and that in the process of action, these peptides adopt an amphipathic α-helical conformation essential to antimicrobial activity [4], [23], [27], [45]. For example, NK-18, a derivative of porcine NK-lysin, displays higher activity compared to its homologous derivatives with low α-helical contents [30]. Given these observations, it is possible that the reduced activity of NKLP22 is due to the shortened α-helical structure contained in this peptide.

Most AMPs are cationic peptides with both hydrophobic and hydrophilic sides, a feature that enables the molecules to be soluble in aqueous environments but also enter into lipid-rich membranes, thus resulting in membrane destruction and killing of the target microorganisms [4], [28], [46], [47]. In our study, PI uptake assay showed that the majority of NKLP27-treated *V. anguillarum* cells were stained positively by PI, suggesting that NKLP27 damaged the membrane integrity of *V. anguillarum*. In agreement with this observation, electron microscopy showed that NKLP27 treatment induced time-dependent destruction of the cells until the cellular structure was completely broken up. These results suggest that the action mechanism of NKLP27 involves initial disruption of the outer membrane of the target bacteria, which eventually leads to loss of intracellular contents and complete collapse of the cell structure.

Recent studies have shown that some AMPs appear to enter into the cytoplasm of target cells and exert antimicrobial activity by interaction with nucleic acids and other target factors. Hsu et al. found that the bovine antimicrobial peptide indolicidin could bind DNA and inhibit DNA sythesis [48]; Sitaram et al. observed an interaction between indolicidin and calmodulin, the latter being involved in the process of DNA synthesis [49]. Likewise, Yan et al. reported that the porcine NK-lysin peptide NK-18 could pierce through cell membrane and accumulate in cytoplasm, where it bound chromosomal DNA [8]. In our study, fluorescence microscopy detected presence of FAM-labeled NKLP27 in the intracellular region of *V. anguillarum*, suggesting that, following disruption of the outer membrane as observed above, NKLP27 must have entered into the cytoplasm of the target cells. However, unlike previous reports, we observed degradation of DNA in NKLP27-treated cells. This observation was corroborated by *in vitro* study, which showed loss of DNA after incubation with NKLP27. These results indicate a likely difference in the action mechanism between NKLP27 and known mammalian AMPs. It is possible that the positively charged residues in NKLP27 enable electrostatic interactions with the negatively charged DNA in the first place; however, the mechanism underlying the subsequent DNA degradation ensued from these interactions has to be investigated.

Consistent with the *in vitro* bactericidal activity observed with NKLP27, *in vivo* study showed that fish administered with NKLP27 exhibited significantly reduced bacterial and viral loads in tissues following *V. anguillarum* and megalocytivirus infection, suggesting that, similar to CsNKL1, NKLP27 exerted antibacterial and antiviral effects under *in vivo* conditions. Although immunoregulatory function has rarely been reported for ATMs, we detected significant upregulation of a number of immune genes in NKLP27-administered fish, suggesting stimulation of immune response by NKLP27. Of these genes, TLR9 and Myd88 are classical signaling molecules that are associated with host immune defense. TLR9 is a type of pathogen-associated molecular patterns (PAMPs) present in eukaryotic cells that recognizes unmethylated CpG sequences in viral and bacterial DNA, and interaction between TLR9 and the microbial ligand leads eventually to Th1-biased cellular and humoral immunity [50]. In mice, TLR9 is known to contribute to antiviral and antibacterial immunity, and TLR9 deletion can cause enhanced gammaherpesvirus pathogenesis and Gram-negative bacterial pneumonia [51], [52]. Myd88 is a universal adapter protein in the signaling pathways of almost all TLRs, including TLR9. Mice with Myd88 deletion exhibited impaired antimicrobial activity and were compromised in both innate and adaptive immunity against acute lymphocytic choriomeningitis virus infection [53], [54]. In addition to TLR9 and Myd88, other upregulated genes induced by NKLP27 include IL-1β and IL-8, which are cytokines that promote proinflammatory response, CsCCK1 and CsCXCe1, which are chemokines that enhance resistance against bacterial infection, and CsISG15, which has been found to facilitate antiviral immunity [38]–[40]. The upregulated expression of all these genes may account at least in part for the ability of NKLP27 to inhibit bacterial and viral infection. A previous report has shown that a human antimicrobial peptide, LL-37, has direct effects on macrophage function by affecting the expression of an array of genes, notably those encoding chemokines and chemokine receptors [55]. Given these findings, it is a possibility that NKLP27 may modulate the activity of immune cells, such as macrophages and lymphocytes, by direct interaction with the cells and result in elevated gene expression as observed with NKLP27-administered fish.

In conclusion, the results of our study demonstrate that NKLP27 is an effective AMP with lytic activity against Gram-negative and Gram-positive bacteria. Like most AMPs, NKLP27 is able to disrupt the outer membrane and penetrate into the cytoplasm of the target cells. However, unlike previously reported AMPs, NKLP27 appears to cause DNA degradation and modulates the innate immune response of the host fish, which very likely contributes to the antibacterial and antiviral capacity of NKLP27. These unique features suggest that NKLP27 is a novel antimicrobial that possibly utilizes an action mechanism differing in some aspects from known ATMs.

## Supporting Information

Figure S1 **Alignment of the amino acid sequences of synthetic peptides derived from fish, chicken, bovine, and porcine NK-lysin.** Dots denote gaps introduced for maximum matching. Numbers in brackets indicate overall sequence identities between NKLP27 and the compared sequences. The consensus residues are in black.
(TIF)
