# Supplementary figures and images for: NKLP27: A Teleost NK-Lysin Peptide that Modulates Immune Response, Induces Degradation of Bacterial DNA, and Inhibits Bacterial and Viral Infection
Source: PLoS One. 2014 Sep 2;9(9):e106543. doi: 10.1371/journal.pone.0106543 (PMC4152322; doi:10.1371/journal.pone.0106543)

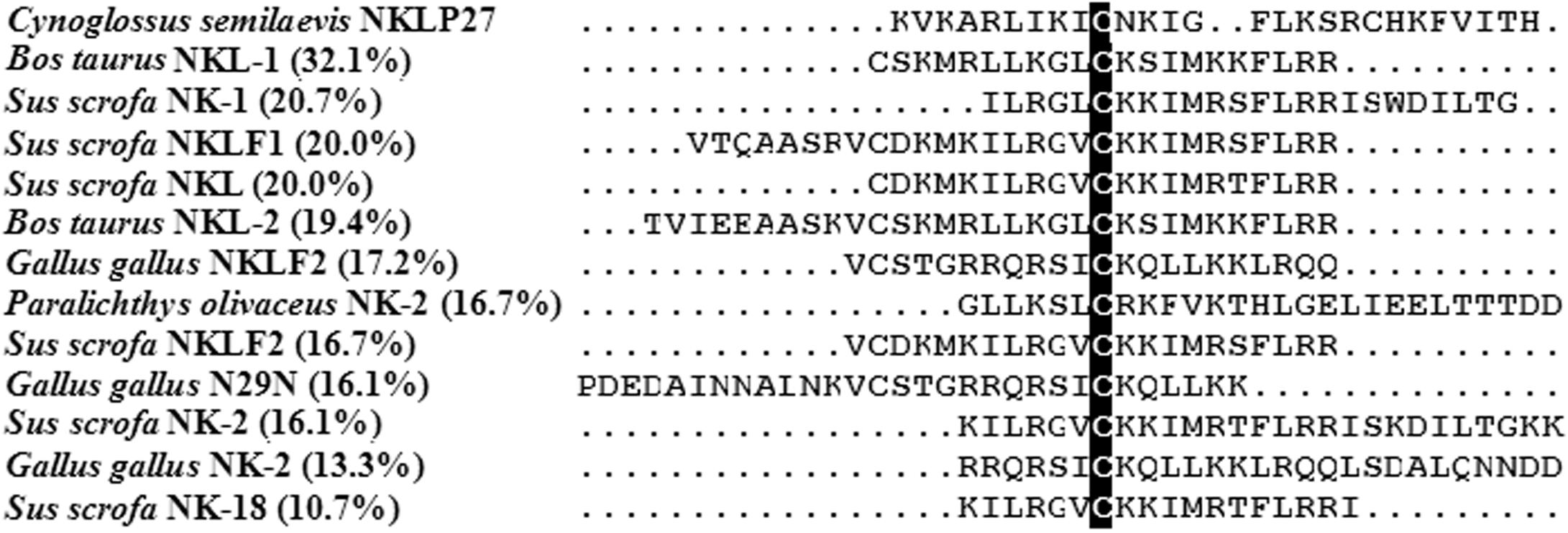

Supplement: Figure S1 — Alignment of the amino acid sequences of synthetic peptides derived from fish, chicken, bovine, and porcine NK-lysin. Dots denote gaps introduced for maximum matching. Numbers in brackets indicate overall sequence identities between NKLP27 and the compared sequences. The consensus residues are in black. (TIF) [file pone.0106543.s001.tif]
